# A large scale mass spectrometry-based histone screening for assessing epigenetic developmental toxicity

**DOI:** 10.1038/s41598-022-05268-x

**Published:** 2022-01-24

**Authors:** Sigrid Verhelst, Bart Van Puyvelde, Sander Willems, Simon Daled, Senne Cornelis, Laura Corveleyn, Ewoud Willems, Dieter Deforce, Laura De Clerck, Maarten Dhaenens

**Affiliations:** 1 ProGenTomics, Laboratory of Pharmaceutical Biotechnology, Ghent University, Ghent, Belgium; 2 Department of Proteomics and Signal Transduction, Max Planck Institute of Biochemistry, 82152 Martinsried, Germany

**Keywords:** Developmental biology, Molecular biology, Stem cells, Health care, Pathogenesis

## Abstract

Toxicoepigenetics is an emerging field that studies the toxicological impact of compounds on protein expression through heritable, non-genetic mechanisms, such as histone post-translational modifications (hPTMs). Due to substantial progress in the large-scale study of hPTMs, integration into the field of toxicology is promising and offers the opportunity to gain novel insights into toxicological phenomena. Moreover, there is a growing demand for high-throughput human-based in vitro assays for toxicity testing, especially for developmental toxicity. Consequently, we developed a mass spectrometry-based proof-of-concept to assess a histone code screening assay capable of simultaneously detecting multiple hPTM-changes in human embryonic stem cells. We first validated the untargeted workflow with valproic acid (VPA), a histone deacetylase inhibitor. These results demonstrate the capability of mapping the hPTM-dynamics, with a general increase in acetylations as an internal control. To illustrate the scalability, a dose–response study was performed on a proof-of-concept library of ten compounds (1) with a known effect on the hPTMs (BIX-01294, 3-Deazaneplanocin A, Trichostatin A, and VPA), (2) classified as highly embryotoxic by the European Centre for the Validation of Alternative Methods (ECVAM) (Methotrexate, and All-trans retinoic acid), (3) classified as non-embryotoxic by ECVAM (Penicillin G), and (4) compounds of abuse with a presumed developmental toxicity (ethanol, caffeine, and nicotine).

## Introduction

In reproductive and developmental toxicity assessment, there is a considerable need for both alternative assays and additional targets^1–3^. This is because (1) it is essentially the most animal-consuming area in drug development and chemical regulatory toxicity testing^4^, (2) interspecies extrapolation of developmental toxicity is not always possible^5^, (3) current testing procedures are relatively time-consuming and resource-intensive^6^, and (4) molecular processes that mediate gene expression during differentiation are still largely understudied. These challenges could be addressed by a high-throughput, but above all more sensitive and human-based in-vitro assay for the detection of developmental toxicity caused by pharmaceuticals and chemicals.

Epigenetic toxicity is a well-known but often neglected phenomenon. It refers to any form of toxicity that is due to alterations in the epigenome, which in turn mediates protein expression and cellular phenotype^7,8^. Four principal epigenetic mechanisms can be distinguished that may contribute to epigenetic toxicity i.e. (1) DNA-methylations, (2) non-coding RNAs, (3) chromatin remodeling, and (4) histone post-translational modifications (hPTMs). These mechanisms are key players in gene expression and especially important in developmental processes such as embryogenesis, X chromosome inactivation, and cell differentiation. Therefore, even minor disturbances in the epigenetic homeostasis during early development may lead to major consequences such as deformations and even cancer or autoimmune and neurological disorders later in life^9,10^.

Recently, hPTMs were found to precede DNA methylation-mediated silencing in the early embryo^11^, illustrating the need for a better understanding of this epigenetic process in developmental toxicity. Briefly, histones are basic and positively charged proteins that form an octamer consisting of two dimers of histone H2A and H2B, and one tetramer of histone H3 and H4 that operate as a central point of attraction for the negatively charged DNA. Accordingly, approximately 147 base pairs of DNA tend to wrap around an octamer resulting in the formation of a nucleosome. Multiple nucleosomes are connected through the linker histone H1 and linker DNA to form chromatin, which is the structural level at which hPTMs play their influential role^12^. In fact, specific hPTMs cause relaxation or reinforcement of the chromatin that leads to transcriptional activation or inhibition, respectively. Ergo, hPTMs may interfere with gene expression by rendering the DNA more or less accessible to transcription factors^13^. A scholarly example of this is histone acetylation, which is associated with transcriptional activation, whereas deacetylation of histones leads to transcriptional repression. This is either caused by the altered biophysical affinity between the histones and DNA (i.e. acetylation reduces the positive charge of the histones), or more importantly, by the recruitment of additional proteins and protein complexes. These proteins, so-called ‘readers’, contain a characteristic domain capable of ‘reading’ a specific hPTM and its stored information^14,15^.

However, this simplistic view of one specific hPTM underlying a biological outcome has long been abandoned and replaced by the concept of the so-called histone code. Herein dozens of hPTMs and the histone variants act together to decide on the final outcome of the chromatin state and its transcriptional activity^15^. In fact, histone modifications are chemical reactions involving energy-rich donors like acyl-CoA (acylations), Adenosine triphosphate (ATP) (phosphorylation), and S-adenosylmethionine (methylation)^16,17^. It is therefore increasingly accepted that histone modification arose as an ancient mechanism to directly sense the energetic state of the eukaryotic cell by translating metabolic information into gene regulation via histones. In turn, this explains the full alphabet of hPTMs that have been discovered to date. Spatially, the PTM combination-centric model even suggests that functionally connected hPTMs can be found on different histone subunits or even on different nucleosomes^15^.

Unfortunately, the widely used antibody-based assays to study hPTMs are confined by a limited number of targets that can be studied in a single experiment and therefore by a lack of combinatorial information. These assays are targeted to a single modification, making screening impossible. Moreover, it is very difficult to find a specific antibody for each modification of interest due to the sequence homology of histone variants and the wide range of hPTMs. As a result, histone antibodies often suffer from cross-reactivity and epitope occlusion^15,18^. In part because of this targeted nature, the large-scale study of the histone code lacks behind compared to other epigenetic mechanisms, which urges for an untargeted screening method to capture the dynamics of the histone code. Fortunately, the introduction of high-end mass spectrometers in the proteomics field enabled the large-scale study of histones and their hPTMs.

We developed a mass spectrometry-based proof-of-concept to develop an assay capable of screening the histone code and hence detecting multiple hPTMs changes simultaneously in response to treatment with compounds of interest. This workflow was applied on human embryonic stem cells (hESCs) treated with compounds from a proof-of-concept library. More specifically, Oct4-reporter hESCs were treated with different concentrations of (1) drugs with a known effect on the hPTMs (BIX-01294 (BIX), 3-Deazaneplanocin A (DZNep), trichostatin A (TSA), valproic acid (VPA), (2) drugs classified as highly embryotoxic by the European Centre for the Validation of Alternative Methods (ECVAM) (methotrexate (MTX) and all-trans retinoic acid (RA)), (3) a drug classified as non-embryotoxic by ECVAM (penicillin G (PenG)), and (4) common substances of abuse with a presumed developmental toxicity (caffeine , ethanol, and nicotine)^19^. Following this dose–response experiment, flow cytometric, Reverse Transcription-quantitative Polymerase Chain Reaction (RT-qPCR), and mass spectrometric (MS) data were acquired to respectively investigate cell death, level of differentiation and the hPTM changes. Accordingly, it is now attainable to detect alterations in hPTMs as an indication of potential toxicity following exposure to a compound of interest.

## Results and discussion

### Experimental design

Recently, we demonstrated that hPTM-changes occur almost instantly, i.e. even down to one hour post-incubation using MS^20^. For this toxicoepigenetic proof-of-principle, we therefore focused on short term effects and opted for 24-h incubation with 4 different concentrations of each compound in the library. Importantly, developmental toxicity affects several cellular processes in embryonic stem cells. More specifically, a compound treatment above a certain concentration can lead to cell death, induce differentiation, result in epigenetic alterations or cause other molecular changes.

**Figure 1:**
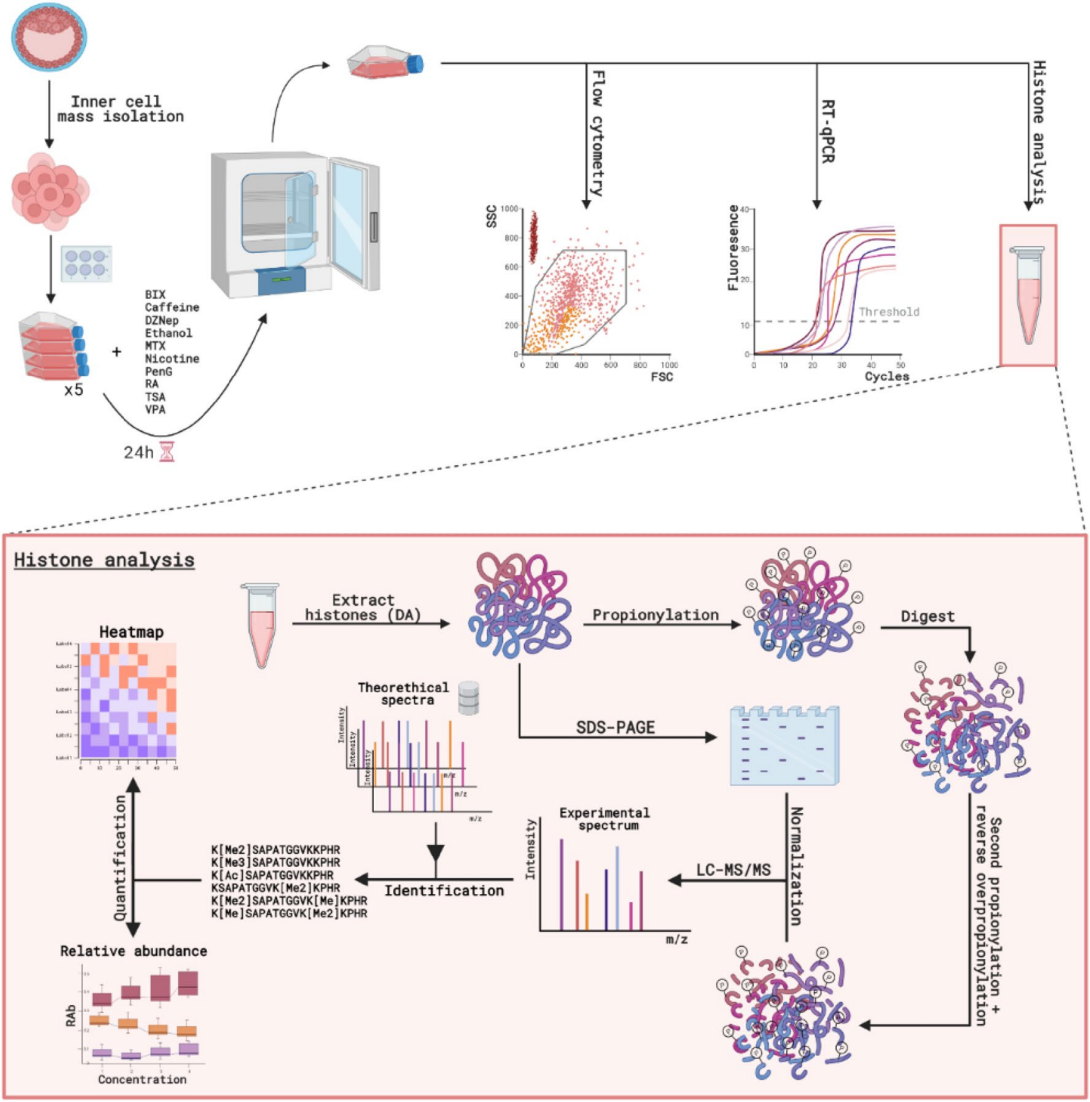
Workflow overview. Commercial human embryonic stem cells (hESCs) were originally derived from an inner cell mass of blastocyst-stage embryos. In this experiment, Oct4-eGFP Knock-In hESCs (WiCell) were cultured in Essential 8 (E8) medium on a precoated feeder-free vitronectin plate. For the baseline culture, hESCs were passaged every 4 to 5 days. After every passage the cells were replated in E8 medium. For the toxicoepigenetic assessment, the medium with test compound was added on day 4. Each compound was added in four different log 10 concentrations with each concentration in quadruplicate. A negative (E8 medium + solvent) and a quality control (E8) were included in respectively quadruplicate and duplicate. After an incubation of 24 h, hESCs were harvested. To perform cell count, and to monitor the number of dead cells and the level of differentiation, flow cytometry was carried out. To monitor differences in the level of gene expression of lineage specification markers (POU5F1, SOX2, Nanog, HAND1 and NES) RT-qPCR was used. Subsequently, the samples were subjected to our MS-based toxicoepigenetic screening of histones. First, the histones were extracted by direct acid (DA) extraction. The amount of extract which corresponds to 400,000 cells was used for quantification by SDS-PAGE to allow normalization against histones alone and to assess the purity of the extract. The remaining extract was subjected to a first propionylation reaction and digested with trypsin, followed by a second propionylation reaction and a reversal of the overpropionylation. Acquisition of the samples was done in randomized batches per compound by using HPLC (capillary flow mode) coupled to MS/MS (DDA-mode). Database searching (Mascot) was performed to identify the peptidoforms present in the samples and was followed by relative quantification on single hPTM-level (RAb-plots) and on peptidoform-level (Heatmap). DA = direct acid; eGFP = Enhanced green fluorescent protein; RT-qPCR = Reverse Transcription quantitative Polymerase Chain Reaction; SDS-PAGE = sodium dodecyl sulfate polyacrylamide gel electrophoresis; HPLC = high-performance liquid chromatography; MS/MS = tandem mass spectrometry; DDA = data-dependent acquisition; RAb = relative abundance. Figure was created with BioRender.com.

A commercial Oct4-eGFP knock-in hESC line expressing eGFP under the control of the POUF1-promoter (Oct4 is encoded by POU5F1), one of the pluripotent markers of hESCs, was selected. This cell line allows simultaneous flow cytometric analysis of cell number (by flow count beads), cell death (visible through PI staining) and cell differentiation (visible through excitation of eGFP). Next, to monitor lineage specification of differentiating stem cells, RT-qPCR was performed to measure gene expression of POU5F1, SOX2, Nanog, HAND1 and NES as markers for respectively pluripotency (first three), mesoderm and early (neuro)ectoderm. Finally, the major focus of the study was the histone analysis of the treated hESCs. Histone analysis requires specific sample preparation (i.e. histone extraction followed by propionylation, digest, an additional propionylation reaction and reversal of nonspecific propionylation). After normalization, through SDS-PAGE, experimental spectra were obtained with LC–MS/MS and matched to theoretical spectra for identification of the peptides present in the sample. This allows to quantify hPTM changes, which we approached in two different ways i.e. through box plots depicting changes in single hPTMs based on RAb and through heatmaps depicting changes in single but also combinatorial hPTMs. Figure 1 summarizes the complete experimental design.

### Proof-of-principle: VPA

Despite its long-standing history in the treatment of epilepsy, migraine and a spectrum of psychiatric disorders, the mechanism of action of VPA is still not entirely elucidated. It is mainly attributed to the increase of gamma aminobutyric acid levels in the brain and the blocking of voltage sensitive channels^21^. However, VPA also has teratogenic properties, for which the underlying molecular mechanism is subject to controversy^22^. Proposed hypotheses include folate antagonism, elevated oxidative stress levels, and interaction with peroxisome proliferator-activated receptors^22,23^. In addition, VPA is an acknowledged histone deacetylases inhibitor (HDACi) which exerts its action on class I and class IIa HDACs resulting in hyperacetylated histones^21^. The teratogenicity was found to be linked to the increase in histone acetylation levels caused by VPA (as well as by TSA, another HDACi), as other VPA- and TSA- analogues without HDAC inhibition capacity were not teratogenic^24^. This makes VPA the prime candidate to demonstrate the applicability of our workflow. Moreover, the reversible nature of this intervention makes epigenetic drugs (epidrugs), like VPA, highly attractive targets in the treatment of a diversity of disorders, e.g. VPA is a promising antitumor agent^8,25^. Irrespectively, also other downstream changes induced in the hPTM homeostasis may trigger toxicity.

hESCs were incubated for 24 h with four different concentrations of VPA (0.04 mM, 0.2 mM, 1 mM, and 5 mM), with each concentration implemented in quadruplicate. A negative control was added by incubating hESCs exclusively in H_2_O (without VPA), since H_2_O was used for dissolving the VPA-samples. A subset of the harvested samples was reserved for flow cytometry and RT-qPCR. The remaining sample was retained for histone analysis.

#### VPA induces (neuro)ectoderm differentiation

**Figure 2:**
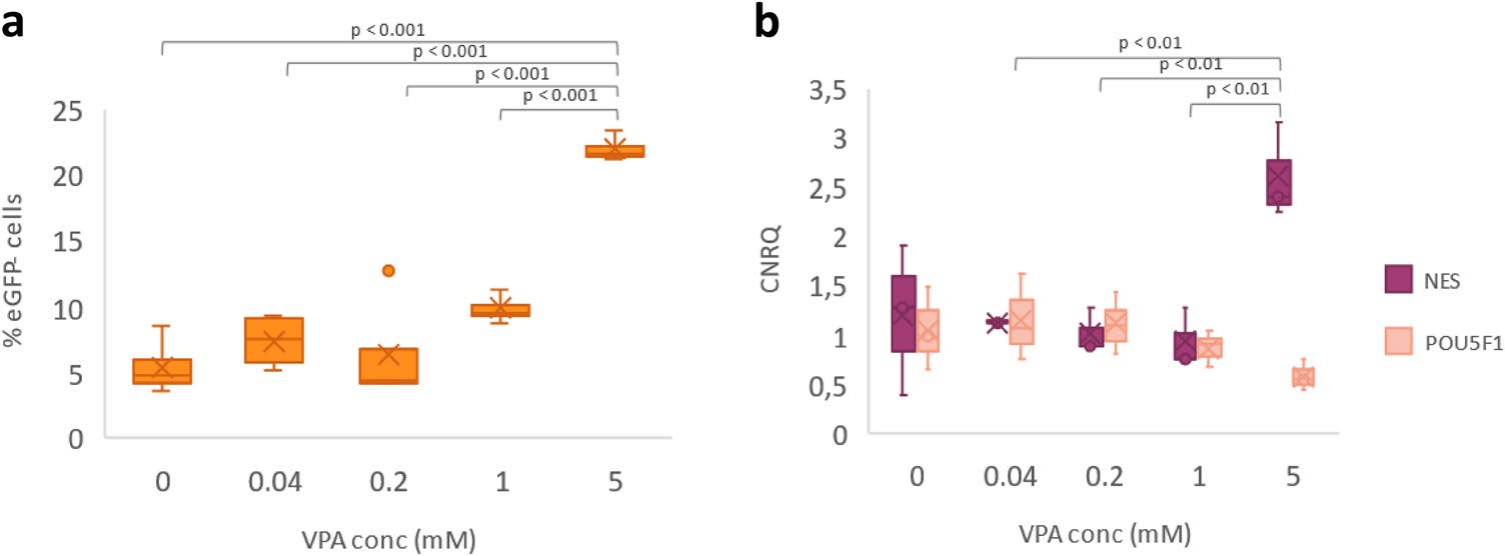
Results of the flow cytometry and RT-qPCR analysis of VPA-treated hESCs. (**a**) The percentage of eGFP negative cells depicted in function of an increasing concentration (in mM) of VPA as determined with flow cytometry. (**b**) The Calibrated Normalized Relative Quantity (CNRQ) of NES and POU5F1, an early (neuro)ectoderm differentiation and a pluripotency marker respectively, represented in function of increasing VPA-concentrations (mM).

Both flow cytometry and RT-qPCR (Fig. 2a,b) analysis indicate that treatment with 1 mM VPA or more results in cell differentiation within 24 h of incubation. An increase in eGFP-negative cells, represents a decrease in Oct4 expression, i.e. a decrease in pluripotency, indicating that the cells are differentiating (Fig. 2a). RT-qPCR was applied for evaluating the expression of pluripotent and lineage specific markers such as POU5F1 and Nestin (NES), respectively. As shown in Fig. 2b, the results are consistent with those reflected by the flow cytometric analysis i.e. a decrease in POU5F1 expression upon increasing concentrations of VPA. For NES, an increased expression is observed, especially at the highest concentration, indicating that the cells start to differentiate towards the (neuro)ectoderm as a result of the VPA treatment^26^.

#### VPA treatment results in a hyperacetylation of histone H3 and H4

**Figure 3:**
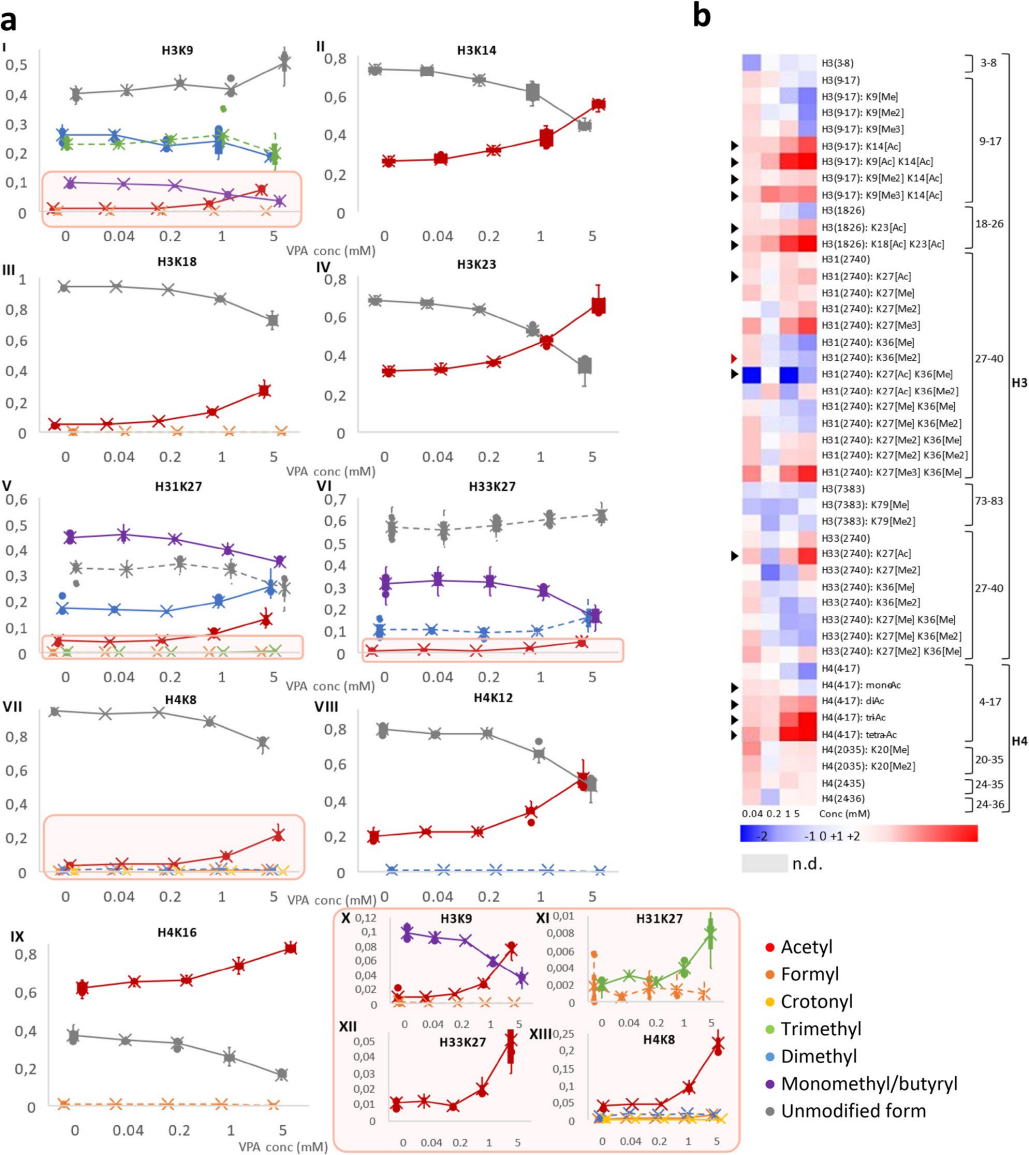
Overview of the VPA-results. (**a**) Relative abundance of all hPTMs competing with acetylation at nine different residues in histone H3 and H4 in function of increasing concentrations of VPA (mM). (I) H3K9, (II) H3K14, (III) H3K18, (IV) H3K23, (V) H31K27, (VI) H33K27, (VII) H4K8, (VIII) H4K12, (IX) H4K16, (X) A zoom with scaled Y-axis of H31K9, (XI) A zoom with scaled Y-axis of H31K27, (XII) A zoom with scaled Y-axis of H33K27, (XIII) A zoom with scaled Y-axis of H4K8. The represented hPTMs are acetyl (red circle), formyl (orange circle), crotonyl (yellow circle), trimethyl (green circle), dimethyl (blue circle), monomethyl/butyryl (violet circle), and the unmodified form (gray circle). hPTMs that are significantly changing i.e. with an ANOVA *P* value < 0.05, between one or more concentrations are depicted by full lines, while dotted lines represent statistically stable hPTMs. Note that histone variants H3.1 and H3.3 can only be distinguished by the peptide H3K27-40. (**b**) Heatmap presenting the changes in hPTMs for a set of peptidoforms, i.e. peptide targets, of histone H3 and H4. Each column represents a different concentration of VPA (from left to right: 0.04 mM, 0.2 mM, 1 mM, and 5 mM). Fold changes were calculated against the solvent (H_2_O) control for the abundances normalized to all histone peptides.

To create a comprehensive picture of the dynamic histone code, we report both the RAb of single hPTMs as well as the peptidoform-centric data, i.e. the measured peptides with their combinatorial hPTMs (Fig. 3). Importantly, the current bottom-up workflow first digests the histones into peptides, giving rise to the different peptidoforms that carry the (combinatorial) hPTMs. In turn this implies that only few peptidoforms can be directly attributed to the exact histone variant they derive from. One exception is the KSAPATGGVKKPHR and KSAPSTGGVKKPHR peptidoforms derived from histone H3.1 and H3.3, respectively. Other peptidoforms should be interpreted without variant connotation. Figure 3a depicts the RAb of all hPTMs competing for nine different acetylated residues. RAb estimates the percentage of the chromatin that is occupied by a given hPTM at a given residue. Importantly, we are able to detect very significant changes in very low hPTM levels occupying below 1% of the chromatin (e.g. Figure 3a: X-XIII). In fact, a significant change of 0.02 to 0.08% was found, for trimethylation of H3K27. The significance of the change shows that it was repeatedly measured, but two important features are not reflected in the RAb metric: (1) not all peptidoforms used in the equation to calculate the RAb ionize as efficiently, which can impact the extent of the change and (2) all low intensity MS measurements, irrespective of histones, are less accurate and show intrinsically higher CVs, making such measurements less accurate^27,28^*.* Irrespectively, small changes will be highly relevant in the context of toxicity testing because localized at promotor or enhancer regions, these changes could induce considerable differences in expression of developmental mediators. The red lines clearly display an overall gain in acetylation levels as the VPA-concentration increases, confirming its action as an HDACi. In general, these acetylations replace other hPTMs, as is shown by e.g. the pronounced decrease in H3K9 methylation levels, which was already established for other HDACis (e.g. TSA)^29^. However, not all other hPTMs decline in response to a rise in acetylation. Most strikingly, dimethylation and trimethylation at H31K27 and dimethylation at H33K27 both rise with their respective acetylated forms, at the cost of monomethylated H31K27 and H33K27. As this is not a known direct enzymatic effect of HDACs, this implies that different histone writers are directly interacting or that cells react to the treatment (toxicity) by altering the activity of other histone writers. We recently showed that in both human and mouse ESCs, H3K27me2/3 is a gatekeeper of pluripotency and that the H4 N-tail is acetylated during differentiation^30^. Therefore, by chemically inducing H4 N-tail acetylation, the cell may increase H3K27 methylations to maintain pluripotency. These downstream or off-target effects will be important in future studies.

Figure 3b shows the different peptidoforms as they are measured by the LC–MS instrument after normalization for sample loading, without the subsequent RAb calculations applied, which can introduce certain biases^20^. When examining the peptidoforms containing acetylations (black arrowheads), the majority increases, especially at higher concentrations of HDACi. Noteworthy, some acetylations are not affected, possibly (1) because of a neighboring PTM blocking enzymatic interaction, (2) because these sites are not a substrate for the histone acetyl transferase (HAT) or HDAC, or (3) because they are an intermediate form that is modified further into a hyperacetylated form at higher VPA concentrations (e.g. H4(4–17): Mono-Ac). Interestingly, an additional internal validation of the performance of the workflow is the opposing trend exhibited by H31K36me2 (Fig. 3b highlighted by red arrowhead) compared to H31K27me3, a recently described direct interaction discovered by using advanced computational models^31^.

In conclusion, this data illustrates the applicability of our untargeted workflow to simultaneously map changes in many different hPTMs.

### Histone fingerprint of the compound library

To illustrate the scalability of the workflow, hESCs were incubated with four different concentrations of in total ten different compounds (VPA included) in quadruplicate each: (1) drugs with a known effect on the histone code (BIX, DZNep, TSA and VPA), (2) drugs that are classified as highly embryotoxic by the ECVAM (MTX and RA), (3) a drug that is classified as non-embryotoxic by ECVAM (PenG), and (4) common substances of abuse with a presumed developmental toxicity (caffeine, ethanol and nicotine). The impact of some of these compounds on specific histone marks has been studied in the past with Chromatin immunoprecipitation sequencing (ChIP-seq)^32–35^. However, ChIP-seq can only obtain information about specific locations in the genome. In contrast, our workflow does not focus on a specific modification site, and therefore can be used complementary to detect targets of interest while also taking combinatorial hPTMs into account.

Again, all cells were monitored for pluripotency and cell death using flow cytometry and RT-qPCR. No other treatment then VPA led to loss of pluripotency of the hESCs within the timeframe of the experiment, i.e. 24 h, as none of the lineage markers significantly changed as a function of concentration for the other compounds (Supplementary Data S2). Nevertheless, due to the highly toxic nature of TSA, an excessive number of cells were dying during incubation at the highest concentration. This was observed by cell detachment from the vitronectin plate, and by flow cytometry after harvest (Supplementary Fig. S2 and Supplementary Data S5). Only 71.6% of the remaining cells were still alive and available for harvest after treatment with 100 nM TSA. This made further histone analysis irrelevant and therefore only three remaining concentrations for TSA were subjected to histone analysis.

**Figure 4:**
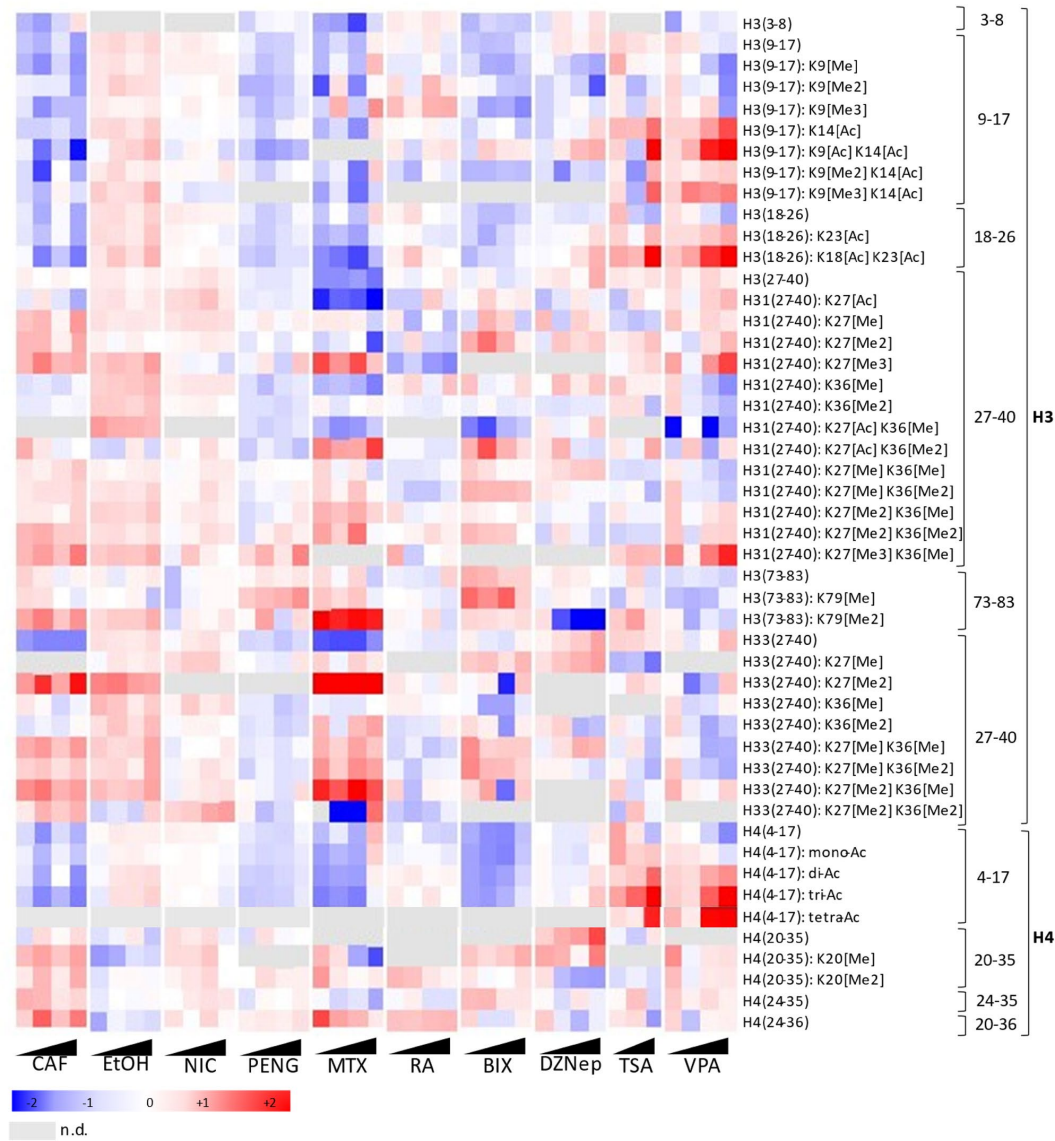
Heatmap representing hPTM fold changes in hESCs treated with increasing concentrations of 10 different compounds. From left to right: hESCs were treated with four increasing concentrations of CAF = caffeine, EtOH = ethanol, NIC = nicotine, PENG = penicillin G, MTX = methotrexate, ATRA = all-trans retinoic acid, BIX = BIX-01294, DZNep = 3-Deazaneplanocin A, TSA = trichostatin A (only three concentrations i.e. 0.1 nM, 1 nM and 10 nM were retained because of excessive cell death in the highest concentration), and VPA = valproic acid. Fold changes were calculated for a set of peptide targets of histone H3 and H4 against the solvent (H_2_O) control for the abundances normalized to all histone peptides. n.d. = not detected.

Figure 4 depicts the fold changes for the increasing concentrations against negative control samples (hESCs incubated in H_2_O or DMSO, depending on the solvent involved) for a set of peptidoforms of H3 and H4.

#### Compounds with a known effect on the histone code

BIX and DZNep are histone methyltransferase inhibitors (HMTis) and TSA and VPA are known HDACis. Note that inhibiting a methyltransferase will reduce methylation, while inhibiting deacetylases will increase acetylation. Indeed, HMTis are the only compounds in which the trimethylation of H31K27 was not observed, in line with the fact that DZNep is capable of inhibiting EZH2, a writer of H3K27me3. Furthermore, DZNep has a known effect on the methylation of H4K20^36^, which was observed as well. Still, a more general inhibition of both repressive and active histone methylation marks was observed as illustrated by the notable decrease in H3K9me2, H3K79me2 and H4K20me2 (Supplementary Fig. S3). Also for BIX, an inhibitor of a G9a histone methyltransferase, our findings are in strong agreement with the literature, since the G9a enzyme is responsible for the methylation of H3K9^37^. Yet a more global effect in methylation is also visible here, as seen by a decrease in H3K27Me2, H3K9Me2 and H3K9Me3, despite the very low concentrations tested (Supplementary Fig. S3). For TSA, a pan-HDAC inhibitor originally known as an antifungal antibiotic, the results are very similar to those already discussed for VPA, with an overall distinct increase in acetylation levels, along with a rise in H31K27me2, H33K27me2, and H31K27me3 and a decrease in methylation of H31K9 and H31K36me2 (Supplementary Fig. S3). Most of these findings are in line with the recently described effects of TSA on mouse ESCs^29^. Moreover, we recently described for the first time that the histone code changes in a very similar way between mouse and human ESC during differentiation, making mouse a potential model for developmental toxicoepigenetics^38^.

In conclusion, for both histone methyl transferase inhibitors BIX and DZNep a general decrease in mainly di- and trimethylation was observed with the most prominent finding being the absence of the trimethyl on the H31K27. For TSA, like for VPA, a general increase in acetylations was detected in accordance with their function as HDACi.

#### Compounds from the ECVAM-classification

The effect of MTX on hPTMs has, to the best of our knowledge, never been investigated, yet we show that this strong embryotoxic compound displays a very prominent and non-coherent dysregulation of the hPTMs. Some hPTMs do not show a concentration-dependence and are heavily affected, even at the lowest concentration (e.g. Figure 4: H31(73–83): K79[Me2] and H33(27–40): K27[Me2]). Therefore, our study could provide a steppingstone to explore the histone fingerprint of MTX more profoundly, for example, by incorporating lower concentrations of MTX or in a time-lapse experimental design. Surprisingly, for ATRA, another strong embryotoxic compound used for the treatment of acne and acute promyelocytic leukemia, the induced changes are much more subtle within the investigated timeframe. The most prominent change is a decrease in H3K27me3, the hallmark of pluripotency, which is in agreement with the ability of ATRA to induce differentiation in ESCs^39^ (Supplementary Fig. S3). The fact that no other lineage specification genes or Oct4 protein change was observed (supplementary Data S2) is in line with the epigenetic role of H3K27me3, which precedes expressional differences. Noteworthy, the hPTM-profile of ATRA is more similar to that of PenG than to MTX, suggesting that the embryotoxicity of ATRA is either (1) not mediated by hPTMs, (2) only emerging at higher concentrations, or (3) a long-term effect that was not sampled in the experimental design. Finally, PenG, a broad-spectrum, beta-lactam antibiotic, was included as a negative control, i.e. not embryotoxic by the ECVAM. No strongly pronounced changes, except for a concentration-dependent effect on K79 monomethylation and a very subtle increase in H4 N-tail acetylation, are observed for PenG in this experimental design (Supplementary Fig. S3).

In conclusion, MTX, a drug that is well-established for its severe embryotoxic status, showed results that are concerning but far from unequivocal in terms of hPTMs. It is clear that MTX exerts an influence on the hPTMs even at low concentrations but the direction in which they are altered is very ambiguous. A more detailed study of MTX is therefore required. For ATRA, another well-documented strong embryotoxic drug the results are much more subtle, which are probably explained by the applied concentrations (1000-fold lower than when used for differentiation of stem cells) and duration of the experiment. PenG, the negative control in terms of embryotoxicity was found to be safe in relation to hPTMs.

#### Compounds of abuse

**Figure 5:**
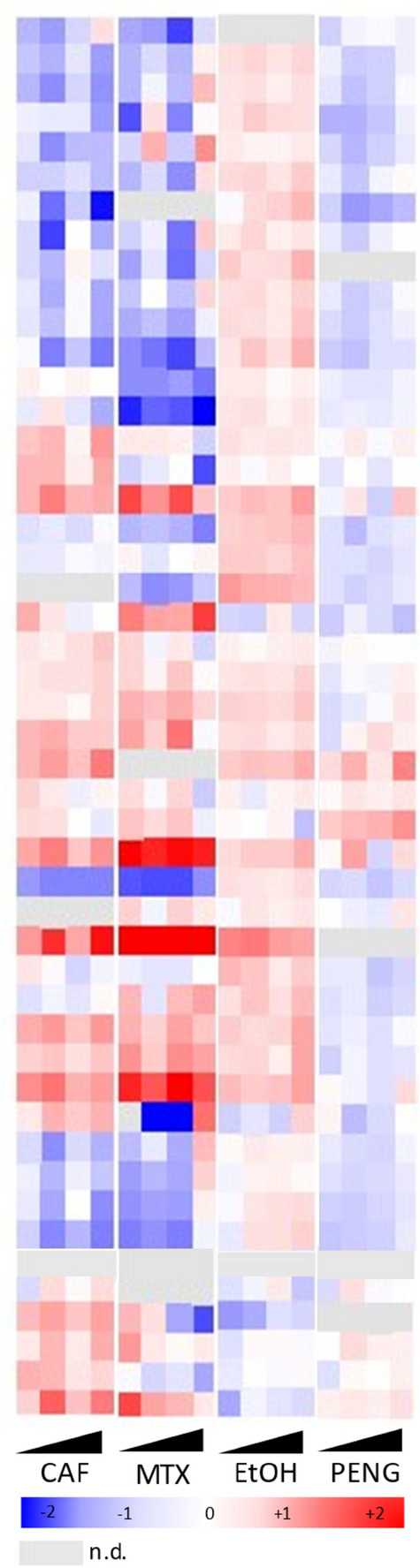
Heatmap highlighting compound clustering between caffeine and methotrexate, as well as ethanol and penicillin G. Fold changes were calculated for a set of peptide targets of histone H3 and H4 against the solvent (H_2_O) control for the abundances normalized to all histone peptides.

Finally, the compounds of abuse displayed relatively moderate fluctuations in their histone signature. Still, caffeine exhibits the most pronounced pattern which, when directly matched, resembles that of MTX most closely (Fig. 5). This is a finding of concern. Currently, it is recommended by the World Health Organization (WHO) not to consume more than 300 mg of caffeine per day during pregnancy because excessive intake may be associated with growth restriction, decreased birth weight, preterm birth or stillbirth^40,41^. Our data suggests that these toxic effects might be linked to changes induced in the histone code. Nevertheless, it should be noted that the metabolization of caffeine was not considered in this experiment. Next, we included ethanol because of its established negative impact during gestation, referred to as fetal alcohol spectrum disorders. Overall, ethanol displays very subtle fold changes, however it does seem to mirror PenG, our negative control in terms of embryotoxicity (Fig. 5).

This suggests that the effect of a one-time intake of ethanol has only a limited influence on the histone code. With several contradictory findings on the effect of ethanol on specific histone marks published earlier, we conclude that more accurate quantification and robust statistical data analysis strategies are required to resolve these very subtle changes in the MS data^32,33,42^. This also holds for nicotine, the addictive compound in cigarette smoke. Smoking is known for its negative impact on pregnancy, e.g. increasing risk of preterm birth, lower birth weight, miscarriage, birth defects, and Sudden Infant Death syndrome^43^. Whereas the toxicity of nicotine has been widely studied, its impact on hPTMs has only been studied in differentiated tissues^44^. Our toxicoepigenetic workflow shows that the hPTM-changes for nicotine are so subtle that it is very conceivable that a one-day intake of nicotine does not affect the hPTMs in stem cells. Again, advanced data analysis strategies need to be developed to make this conclusion more founded.

In conclusion, for the compounds of abuse, caffeine had the most pronounced profile which has some similarities to the profile of MTX, albeit much less pronounced. However, this can be considered alarming but requires more in-depth investigation. For both ethanol and nicotine, the results show that single ingestion during pregnancy does not produce major effects on the hPTMs.

### Future perspectives

To date, little is known about the effects of different compounds on the hPTM-landscape. Yet, our comprehensive overview of the hPTM changes induced by ten compounds in stem cells shows that most compounds have a (subtle) effect on the histone code.

Our study is the ideal steppingstone to extend the knowledge on this form of epigenetic toxicity in light of developmental toxicity. This can be done by (1) including other compounds of interest, (2) adjustment of dose, (3) adjustment of incubation time or the use of time-lapse experimental designs and, (4) developing more advanced statistical methods and algorithms to cluster compounds to facilitate the decision-making toolbox. Moreover, the applicability of our workflow goes far beyond developmental toxicity. Firstly, other forms of toxicity can also be investigated depending on the cell line used, e.g. hepato-and nephrotoxicity by using liver and kidney cells respectively. Investigating other cell lines and models will also be of great importance to find out any differences from the stem cell model. It is possible that certain effects on the hPTMs are not expressed in the hESCs but will be expressed in another model. Secondly, this study is not only important in the context of toxicoepigenetics but is also a promising tool in the field of pharmacoepigenetics. As these epigenetic modifications are interesting targets due to their dynamic and reversible character, the development of epidrugs is gaining momentum. Especially in oncology, the use of epidrugs is on the rise and our workflow may contribute to discovering or elucidating the mechanism of action of these drugs^45,46^. Moreover, personalized medicine is receiving growing attention and this study can contribute to this as well^47^. For example, it is possible to determine whether a patient exhibits a particular hPTM characteristic on which the drug will act, thereby predicting whether or not the treatment is likely to succeed. Finally, the scope of this study can be extended outside the pharmaceutical context including applications for environmental toxicity and food safety.

However, to make the results of this workflow easier to interpret, more reliable and consequently easier to implement, we are still working on some improvements both in terms of acquisition and data analysis. For instance, with our current LC–MS/MS settings, it is difficult to acquire modified forms of H3K4. There are two reasons for this: (1) this PTM site is located on a small tryptic peptide, that consequently elutes early, making it difficult to analyze and, (2) our mobile phase contains DMSO, which improves ionization, but also causes charge state reduction. Therefore, the H3K4 peptide occurs mostly as a singly charged ion^48^. Nevertheless, this modification site can be of interest as methylation of H3K4 is associated with active transcription. Consequently, besides optimization of the LC gradient, acquisition parameters can be adjusted to also target singly charged precursors or DMSO can be removed from the mobile phase to include modifications of H3K4 in the future. These improvements in LC–MS/MS settings should also result in a better separation of other peptides, which in turn will allow more accurate quantification, so that differences will become even more apparent. Note that the effect of withdrawing DMSO on the other histone peptides should be assessed as well, since it is known that doubly charged peptides are best annotated as they mostly generate singly charged fragments. Furthermore, including data-independent acquisition technologies like (Scanning) SWATH will result in an improved quantification and will be a stepping stone in the transition towards a multiple or parallel reaction monitoring, respectively MRM and PRM, assay^20^. When focusing on data-analysis, we already mentioned that caution is always required when reporting RAbs^20^. Depending on the peptidoforms used for the calculation in combination with the ionization effects, RAbs can lead to a confusing and even misleading form of reporting. Therefore, we are working on more advanced statistical approaches that could contribute to better reporting and consequently a better understanding of the outcomes.

In conclusion, we demonstrated that with our workflow toxicoepigenetic screening on histones is feasible and will yield very rich data, for which more streamlined interpretation tools are yet to be developed. Integration of this epigenetic information into the field of toxicology is a promising addition that offers an opportunity to gain novel insights into toxicological phenomena^10^. We envision a future wherein 100–200 histone peptidoforms are brought together in a single MRM or PRM assay that runs in < 10 min per sample, enabling 6 samples per hour or nearly 150 samples per day per instrument, which get automatically analyzed to create a user-friendly report. Storing all results in a central database will finally allow to cluster novel compounds with other, known toxicoepigenetic effects, classifying them according to potential toxicity level in a given targeted cell type. As a result, this proof-of-concept to develop a screening assay can contribute to the (safe) development of drugs as well as to the field of environmental toxicity and food safety.

## Materials and methods

### Cell culture and harvest

Oct4-enhanced green fluorescent protein (eGFP) knock-in hESCs (WA01, H1, WiCell Research Institute, NIH Registration Number 0043) were used in this study. This hESC line has a normal 46, XY karyotype, O + blood type. Karyotype analysis was done at the start and end of the experiment. Cells were cultured in Essential 8 (E8) medium on a precoated Vitronectin XF™ plate (0.5 μg/cm^2^, Primorigen) in 5% O2, 5% CO2 and 37 °C. Cells were routinely passaged with 0.5 mM Ethylenediaminetetraacetic acid (EDTA) in Dulbecco's phosphate-buffered saline (DPBS) according to the manufacturer’s protocol of culturing hESCs in E8 medium. After every passage, the cells were replated in E8 medium; on day 4, the medium containing the test compound was added. Each compound was added in four different concentrations and a negative- and quality control were included (Table 1). The concentration range of the components was determined based on already published data and/or known circulating blood concentrations. The references on which these concentrations are based can be found in Table 1. Since it was less straightforward for the substances of abuse to determine concentrations, we provide some additional info on which these choices rely. First, blood nicotine concentrations in the range of 0.06 to 0.3 µM indicates the subject is either actively using a tobacco product or on nicotine replacement therapy. A tobacco user after 2 weeks of complete abstinence has the same level of serum nicotine concentration (< 0.02 µM) as a nontobacco users^49,50^. Second, for ethanol the first effects appear at a blood alcohol level of 0.01% (2.17 mM), such as relaxation and mild euphoria. At a blood alcohol level of 0.3% (65 mM) and higher, complete loss of consciousness may occur and a blood alcohol level of 0.5% (109 mM)and higher may even cause death^51^. Lastly, plasma caffeine levels are usually in the range of 2–10 mg/L (_~_10–50 µM) in coffee drinkers. In general, toxicological symptoms often begin above concentrations of 15 mg/L (~ 75 µM), a concentration of 50 mg/L (~ 250 µM) is considered “toxic” and concentrations of 80 mg/L (~ 400 µM) or greater are considered lethal^52,53^.

**Table 1:** Overview of the included compounds with respectively the applied solvent and the four concentrations.

Compound (unit)	Solvent (%) in E8	Concentration			
		1	2	3	4
PenG (µM)^54–56^	H_2_O (2%)	5	50	500	5000
VPA (mM)^55–62^	H_2_O (0.01%)	0.04	0.2	1	5
ATRA (nM)^55,56,59,62–64^	DMSO (0.01%)	0.2	2	20	200
MTX (µM)^56,62,65,66^	DMSO (0.12%)	0.1	1	10	100
TSA (nM)^60,61^	DMSO (0.01%)	0.1	1	10	100
BIX (nM)^37,57^	H_2_O (0.4%)	0.01	0.1	1	10
DZNep (µM)^67,68^	H_2_O (0.4%)	0.01	0.1	1	10
Ethanol (mM)^51,69^	Already a solution	0.1	1	10	100
Caffeine (µM)^52–54,56^	H_2_O (1%)	1	10	100	500
Nicotine (µM)^49,50,70^	H_2_O (0.4%)	0.002	0.02	0.2	2

After an incubation of 24 h, the cells were harvested. Briefly, cells were washed in PBS and incubated for 5 min at 37 °C in 2.5 mL Trypsin–EDTA (0.05%). Subsequently, 2.5 mL trypsin-inhibitor was added, and the cells were dissociated. 150 μL of the suspension was transferred to an Eppendorf tube for subsequent flow cytometric analysis. 500.000 cells were isolated for mRNA expression studies. The remaining cells were frozen as a dry pellet in liquid nitrogen for histone extraction. Each concentration and the negative controls were conducted in fourfold, while the quality controls were conducted in twofold. Karyotype analysis was performed at the beginning and end of the experiment, indicating that the cells maintained normal karyotypes throughout the study (data not shown). The culture was free of mycoplasma contamination (data not shown).

### Flow cytometry

Cell count and -viability were assessed using flow cytometry. Before the analysis, cells were resuspended in PBS + 1% bovine serum albumin (BSA) solution. Propidium iodide (PI) (Sigma-Aldrich) was added to measure cell viability. Flow count beads (Analis) were added to acquire absolute cell counts. The samples were analyzed using Beckman Coulter Cytomics FC500 and CXP analysis software. A minimum of 10.000 events was acquired for each sample. Data analysis was done using the Kaluza analysis software (Beckman Coulter Life Sciences). The different gates are depicted in Supplementary Fig. S1.

### RT-qPCR

RNA isolation and RNA quality assessment were performed as described previously^71^. Briefly, 500.000 cells were resuspended in TRIzol (Invitrogen) and stored at − 80 °C. For RNA isolation, chloroform was added to the thawed samples, with subsequent phase separation and purification using an RNeasy Mini kit (Qiagen). After DNase treatment (Qiagen) and a washing step, RNA was eluted. Samples were stored at − 80 °C. RNA quality was assessed using the RNA 6000 Nano Kit (Agilent). RNA was quantified using a RiboGreen assay. Complementary DNA (cDNA) was synthesized using The High-Capacity RNA-to-cDNA™ Kit (Thermo Fischer Scientific) according to the manufacturer’s protocol and subsequently stored at − 20 °C. RT-qPCR was performed using the LightCycler 480 (Roche). For each reaction 1 μl of cDNA (2 ng/μl) was mixed with 10 μl of the LightCycler® 480 SYBR Green I Master (Roche) in a 384-well plate. Cycling conditions were initial denaturation at 95 °C for 5 min followed by 45 cycles of 95 °C for 10 s, 57 °C for 20 s, and 72 °C for 20 s. A subsequent heating step from 40 °C to 95 °C was added to obtain melting curves. The primer sequences for the housekeeping genes, B2M (ID #2) and RPL13A (ID #6) (final concentration 300 nM), are available in the RTPrimerDB database. The included primers are listed in Table 2. Relative quantification of the markers was calculated using the qbasePLUS software. Each sample is relative to a calibrator, in this case untreated hESCs (negative control), and was normalized for two reference loci: B2M and RPL13A. For each marker, statistical analysis was performed using a One-way ANOVA test.

**Table 2:** Sequence of the forward and the reverse primer of the genes that were tested.

Gene	Sequence of forward primer (5′-3′)	Sequence of reverse primer (5′-3′)	Concentration (µM)
Sox-2^72^	AGTCTCCAAGCGACGAAAAA	TTTCACGTTTGCAACTGTCC	2
Nanog^73^	CCAACATCCTGAACCTCAGC	TGCTATTCTTCGGCCAGTTG	1
POU5F1^74^	GAGGAGTCCCAGGACATCAA	AATAGAACCCCCAGGGTGAG	1
HAND1^75^	CCTATCTGGCTCTTTCTCTCTTGTC	CATCTTCCTGCGTCTGGTTCTC	1
NES^76^	GAAACAGCCATAGAGGGCAAA	TGGTTTTCCAGAGTCTTCAGTGA	1

### Histone extraction, propionylation and digestion

As each sample was harvested from a different culture flask, cell count could considerably differ between samples treated with the same compound. To minimize variation, samples were split up into technical replicates, such that each sample contained the same number of cells. For each component, sample cell count was normalized to the lowest cell count sample. This resulted in a total of 258 experimental samples, on which histone extraction and propionylation were performed as previously described^77,78^. Briefly, the cell pellet was resuspended in 0.4 N hydrogen chloride (HCl) and incubated for 4 h on a rotator at 4 °C. The histones were precipitated with 33% trichloroacetic acid (TCA) on ice for 30 min. The amount of extract corresponding to 400,000 cells was used for histone quantification by sodium dodecyl sulfate polyacrylamide gel electrophoresis (SDS-PAGE) on a 18% TGX gel (Biorad). The remaining purified histones were dissolved in 20 µL 1 M triethylammonium bicarbonate (TEAB) buffer, pH 8.5. Next, 20 μL of propionylation reagent (propionic anhydride: 2-propanol 1:79 (v/v)) was added, for an incubation of 30 min at room temperature. This was followed by adding 20 µl MilliQ water for 30 min at 37 °C. The histone samples were digested overnight at 37 °C using trypsin (Promega) (at an enzyme/histone ratio of 1:20 (m/m)) in 500 mM TEAB, supplemented with calcium chloride (CaCl_2_) and acetonitrile (ACN) to a final concentration of 1.0 mM and 5% respectively. Subsequently, the derivatization reaction was carried out again to cap peptide N-termini. Aspecific overpropionylation at serine (S), threonine (T) and tyrosine (Y) was reversed by incubating the samples in 50 µL 0.5 M hydroxylamine and 15 µL ammonium hydroxide for 20 min at room temperature followed by adding 30 µl of 100% formic acid (FA).

### Liquid chromatography and mass spectrometry analysis

Because of the size of the experiment, the samples were run in batches per compound. Within each batch, the samples were analyzed in a randomized fashion by liquid-chromatography coupled with tandem MS (LC–MS/MS). Therefore, the propionylated peptides were resuspended in 0.1% FA to ensure that a 5 μl injection resulted in 2 μg of histones and 50 fmol of Beta-Galactosidase (ß-gal) internal standard on-column. Peptides were trapped on a Triart C18 column (5 × 0.5 mm, YMC) and separation was performed using a Triart C18 column (150 × 0.3 mm, YMC) on a NanoLC 425 system operating in capillary flow mode (5 μl/min). The mobile phase consisted of 0,1% FA in water supplemented with 3% dimethyl sulfoxide (DMSO) (Buffer A) and 0,1% FA in ACN (Buffer B). A low pH reversed phase 60 min gradient going from 3%–45% Buffer B was used, with a total run time of 86 min per sample. The sample list was interspersed with propionylated bovine histone standards (Roche) for alignment. Calibration and monitoring of the LC–MS/MS system was done respectively by incorporating ß-gal internal standard runs every five samples and *E. coli* Auto-QC samples at the beginning, middle and end of every batch. Data-dependent acquisition (DDA) was executed on a TripleTOF 5600 (AB Sciex) operating in positive mode, acquiring full scan MS1 (m/z 400–1250) and MS2 spectra (m/z 65–2000, high sensitivity mode) with a scan time of 250 and 200 ms respectively. For the MS2 spectra, a rolling collision energy with a spread of 15 V was applied and a maximum of 10 precursors (charge state + 2 to + 5) exceeding 300 cps were isolated for fragmentation followed by an exclusion for 10 s. Targeting 10–12 data points per LC-peak, the cycle time was set at 2.3 s.

### Data analysis

Mass spectrometric data analysis was performed as previously described^79^ yet some modifications were implemented. For every compound, raw data from all runs were imported in a single experiment and all runs were aligned against a bovine histone standard in Progenesis QIP 4.2.7 (Nonlinear Dynamics, Waters https://www.waters.com/waters/en_US/Progenesis-QI-for-Proteomics/nav.htm?cid=134790665&locale=en_US). Next, feature detection was performed on the samples excluding the bovine histone samples to eliminate features that are only present in the bovine histones and not in the hESCs samples. The twenty MS/MS spectra closest to the elution apex were selected for each precursor ion and merged into a single ∗ .mgf file. On this file, two types of searches in Mascot (Matrix Science) were performed. Therefore, the experimental MS/MS-spectra were compared to theoretical spectra obtained after in silico digest of the appropriate protein database, resulting in a given score for each peptide, which enabled (1) a quality search to identify non-propionylated standards (ß-gal) and to assess the amount of over- and underpropionylation, which was acceptable (data not shown), and (2) an error tolerant search to identify the proteins present in the sample. For both searches, the following parameters were included: (1) mass error tolerances for the precursor ions and its fragment ions were set at 10 ppm and 50 ppm respectively; (2) enzyme specificity was set to Arg-C, allowing for up to one missed cleavage; (3) variable modifications included N-terminal propionylation and propionylation on K for the quality search and deamidation on asparagine (N) and glutamine (Q) and oxidation of methionine (M) for the error tolerant search, (4) no fixed modifications were included for the quality search and N-terminal propionylation and propionylation on K were set as fixed modifications for the error tolerant search; and (5) a complete Human SwissProt database (downloaded from UniProt and supplemented with contaminants from the common Repository for Adventitious Proteins (cRAP) database (https://www.thegpm.org/crap/)) was used. Based on the error tolerant search, a FASTA-database was generated, and a fixed hPTM set was determined for all 10 compounds for further analysis (i.e. based on the highest ranked hPTMs in the error tolerant searches for each compound, together with the biologically most commonly studied hPTMs (acetylations and methylations)). Next, a second ∗ .mgf file containing the three MS/MS spectra closest to the elution apex per feature was exported to perform a Mascot-search with the following parameters: (1) mass error tolerances for the precursor ions and its fragment ions were set at 10 ppm and 50 ppm respectively; (2) enzyme specificity was set to Arg-C, allowing for up to one missed cleavage site; (3) variable modifications included acetylation, butyrylation, crotonylation, trimethylation and formylation on K, methylation on R, dimethylation on both K and R, deamidation on N, Q and R and oxidation of M; and (4) N-terminal propionylation and propionylation on K were set as fixed modifications. Database searching was performed against the above mentioned custom-made FASTA-database. The Mascot result files (∗ .xml-format) were again imported into Progenesis QIP 4.2.7 for annotation. Features that were annotated as peptidoforms derived from histones were manually validated and curated by an expert to resolve isobaric near-coelution. Normalization of the samples (e.g. to correct for different sample loading) was performed against all histone peptides. This is important, because the workflow aims at quantifying changes in the hPTMs, not in the expression of the histones themselves. Outlier detection and removal was based on normalization on two levels: (1) Before identification, when normalization is still done against all detected precursor ions, a normalization factor greater than 10 was used to filter out under-loaded samples (this was only the case for replicate 001C and 01B2 of DZNep), and (2) After identification, when normalization is done against all histone peptides, an estimated standard deviation (~ STD) greater than 0.4 was used to filter out samples with too much internal variation. Progenesis QIP 4.2.7 uses ratiometric data in log space, along with a median and mean absolute deviation outlier filtering approach to calculate the estimated standard deviation (~ STD) and normalization factor (Supplementary Data S1). Finally, the deconvoluted peptide ion data for every experiment (i.e. for each component separately) was exported from Progenesis QIP 4.2.7 for further analysis (Supplementary Data S2). The mass spectrometry proteomics data have been deposited to the ProteomeXchange Consortium via the PRIDE partner repository with the dataset identifier: PXD026468 and 10.6019/PXD026468.

Heatmaps of both VPA and all 10 compounds together were generated using Qlucore Omics Explorer (3.6) for a predefined set of target peptides. For every compound, averages of the normalized abundances were calculated per concentration and the log fold changes were determined for every concentration towards the negative control: $\log_{2} \left( {\frac{{average\,\, conc_{x} }}{average \,\,neg}} \right)$ (Supplementary Data S3). For VPA, relative abundances (RAb) were calculated as previously described^20^ by dividing the area under the curve (AUC) for each peptidoform containing the considered hPTM by the sum of the AUCs for all observed forms of that peptide $\frac{{\sum (intensities\,\, of\,\, peptidoforms \,\,containing \,\,hPTM _{x} )}}{{\sum \left( {intensities\,\, of \,all\,\, peptidoforms} \right)}}$ (Supplementary Data S4). Visualization of the RAb is performed via box plots with the median included for the quartile calculation. For each hPTM, an ANOVA test and a paired t-Test between each concentration was accomplished to determine which concentrations introduced a significant difference in the RAb of each individual hPTM (Supplementary Data S6).

## Data availability

Data have been deposited to the ProteomeXchange Consortium via the PRIDE partner repository with the dataset identifier PXD026468 and 10.6019/PXD026468 (Reviewer account details to access the data: Username: reviewer_pxd026468@ebi.ac.uk & Password: BOPqFFQJ).

## Supplementary Information

Supplementary Information 1.

Supplementary Information 2.

Supplementary Information 3.

Supplementary Information 4.

Supplementary Information 5.

Supplementary Information 6.

Supplementary Information 7.

## Abbreviations

Ac: Acetylation
ACN: Acetonitrile
ATP: Adenosine triphosphate
ATRA: All-trans retinoic acid
AUC: Area under the curve
ß-gal: Beta-galactosidase
BIX: BIX-01294
CaCl_2_: Calcium chloride
cDNA: Complementary DNA
ChIP-seq: Chromatin immunoprecipitation sequencing
DDA: Data-dependent acquisition
DMSO: Dimethyl sulfoxide
DPBS: Dulbecco's phosphate-buffered saline
DZNep: 3-Deazaneplanocin A
ECVAM: European Centre for the Validation of Alternative Methods
EDTA: Ethylenediaminetetraacetic acid
eGFP: Enhanced green fluorescent protein
Epidrugs: Epigenetic drugs
E8: Essential 8
FA: Formic acid
HAT: Histone acetyltransferase
HDACi: Histone deacetylase inhibitor
hESC: Human embryonic stem cell
HMTi: Histone methyltransferase inhibitor
HPLC: High-performance liquid chromatography
hPTM: Histone post-translational modification
HCl: Hydrogen chloride
K: Lysine
M: Methionine
Me: Monomethyl
Me2: Dimethyl
Me3: Trimethyl
MS: Mass spectrometry
MS2 or MS/MS: Tandem mass spectrometry
MTX: Methotrexate
N: Asparagine
NES: Nestin
PBS: Phosphate buffered saline
PenG: Penicillin G
PI: Propidium iodide
Q: Glutamine
R: Arginine
RAb: Relative abundance
RT-qPCR: Reverse transcription-quantitative polymerase chain reaction
S: Serine
SDS-PAGE: Sodium dodecyl sulfate polyacrylamide gel electrophoresis
T: Threonine
TCA: Trichloroacetic acid
TEAB: Triethylammonium bicarbonate
TSA: Trichostatin A
VPA: Valproic acid
WHO: World Health Organization
Y: Tyrosine
