# Supplementary material for: A large scale mass spectrometry-based histone screening for assessing epigenetic developmental toxicity
Source: Sci Rep. 2022 Jan 24;12:1256. doi: 10.1038/s41598-022-05268-x (PMC8786925; doi:10.1038/s41598-022-05268-x)
Supplement: Supplementary file 7 — Supplementary Information 7. [file 41598_2022_5268_MOESM7_ESM.pdf]

Supporting information

A LARGE SCALE MASS SPECTROMETRY-BASED HISTONE SCREENING FOR ASSESSING EPIGENETIC DEVELOPMENTAL TOXICITY

Sigrid Verhelst[1], Bart Van Puyvelde[1], Sander Willems[2], Simon Daled[1], Senne Cornelis[1], Laura Corveleyn[1], Ewoud Willems[1], Dieter Deforce[1], Laura De Clerck[1,#], Maarten Dhaenens[1,#,*]

[1] ProGenTomics, Laboratory of Pharmaceutical Biotechnology, Ghent University, Ghent, Belgium
[2] Department of Proteomics and Signal Transduction, Max Planck Institute of Biochemistry, 82152 Martinsried, Germany
[#] These authors jointly supervised
* Corresponding author

Table of Contents

Supporting Data 1
Data S1 1
Data S2. 1
Data S3. 1
Data S4. 1
Data S5. 1
Data S6. 1
Supporting Figures 2
Figure S1. 2
Figure S2. 3
Figure S3. 5

Supporting Data

Data S1. Excel report containing the calculations for the median, count, median of the absolute deviation, estimate of the standard deviation, and the robust estimation mean and limits for the purposes of normalization.

Data S2. Excel report containing the deconvoluted peptide ion data exported from Progenesis QI for proteomics for all the samples included in the experiment.

Data S3. Excel report of the data used for the creation of the heatmap for the example of VPA. The same workflow was used for all other compounds.

Data S4. Excel report containing the calculations of the relative abundance and the boxplot representation for valproic acid. The same workflow was used for all other compounds.

Data S5. Excel report of the flow cytometry data exported from the Kaluza analysis software (Beckman Coulter Life Sciences) and CNRQ values exported from the qbasePLUS software.

Data S6. Excel report containing the ANOVA values and the results of the pairwise comparisons of the t-tests for the VPA-results.

Supporting Figures

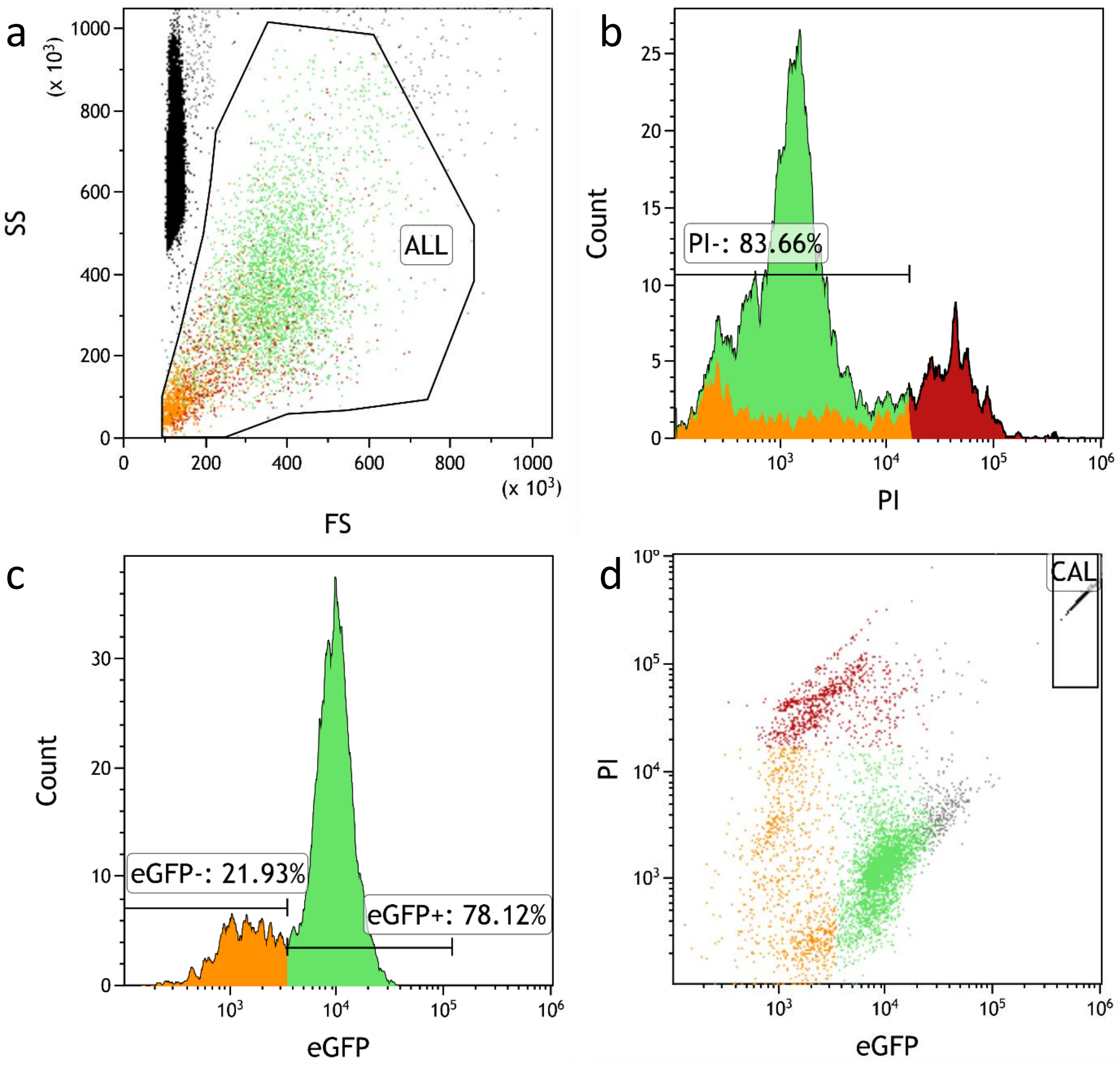

Figure S1. Flow cytometry gates. (**a**) The side scatter (SS) in function of the forward scatter (FS). The black dots on the left represent the flow count beads. The green dots are the living cells (PI-), the red dots are the dead cells (PI+), and the orange dots are the eGFP negative cells. (**b**) is gated on A and is used to select the living cells (PI-). (**c**) is gated on the PI- cells and is used to select the differentiating cells (eGFP-). (**d**) PI in function of eGFP. In the right upper corner the flow count beads are represented. The grey dots are the cells that were not gated. (This is sample VPA 5D)

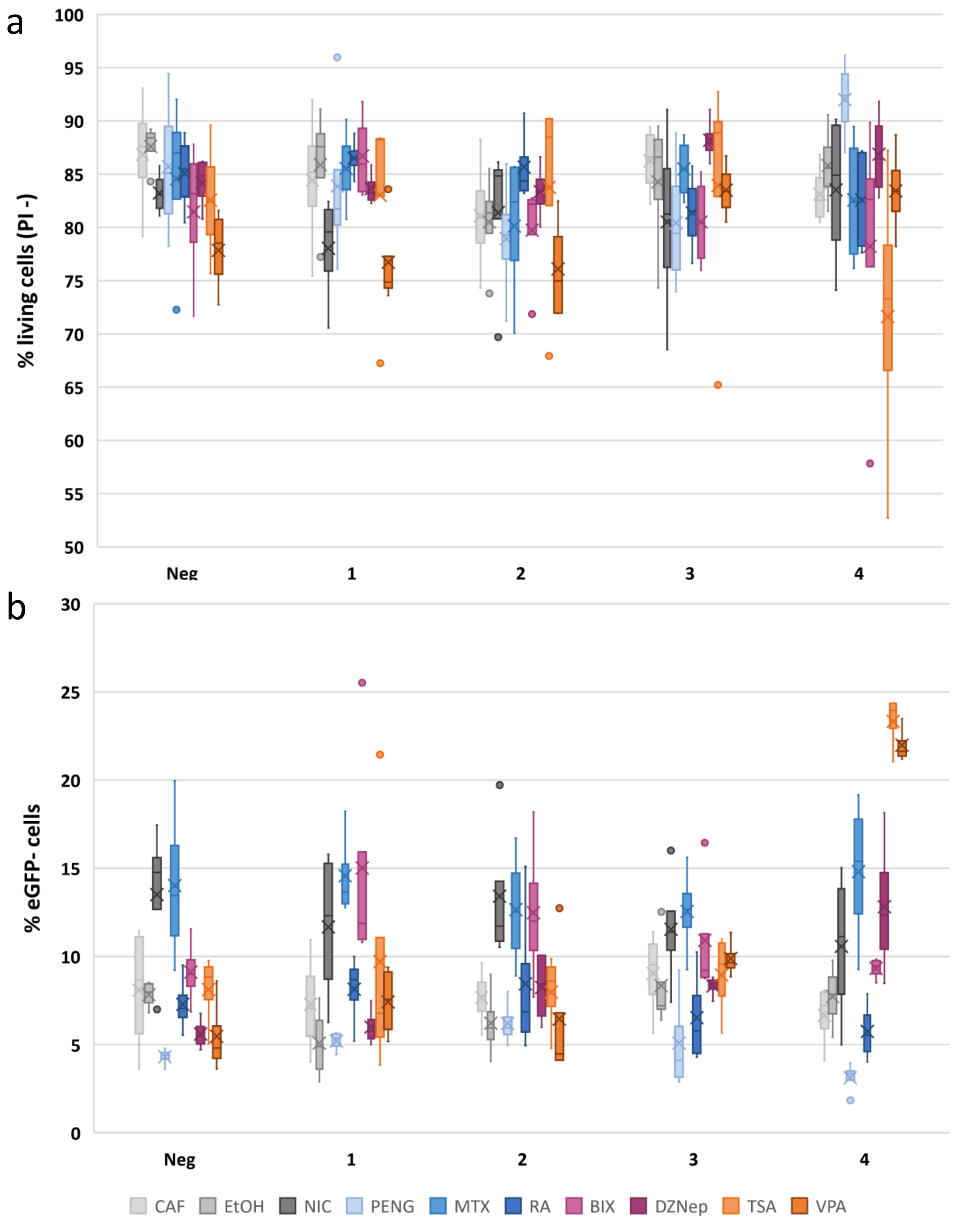

Figure S2. Flow cytometry data of eGFP reporter hESC. (**a**) shows the percentage of the living cells (PI-). PI was used for staining of dead cells (PI+). The highest concentration of TSA resulted in more cell death. (**b**) shows the percentage of eGFP positive cells within the living cells. The highest concentration of VPA and TSA resulted in an increase of eGFP+ cells, i.e. differentiating cells.

BIX

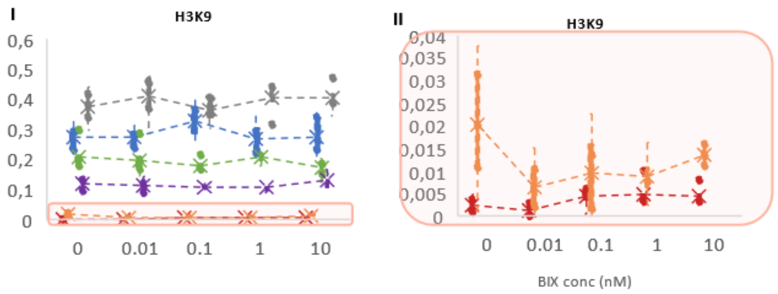

DZNep

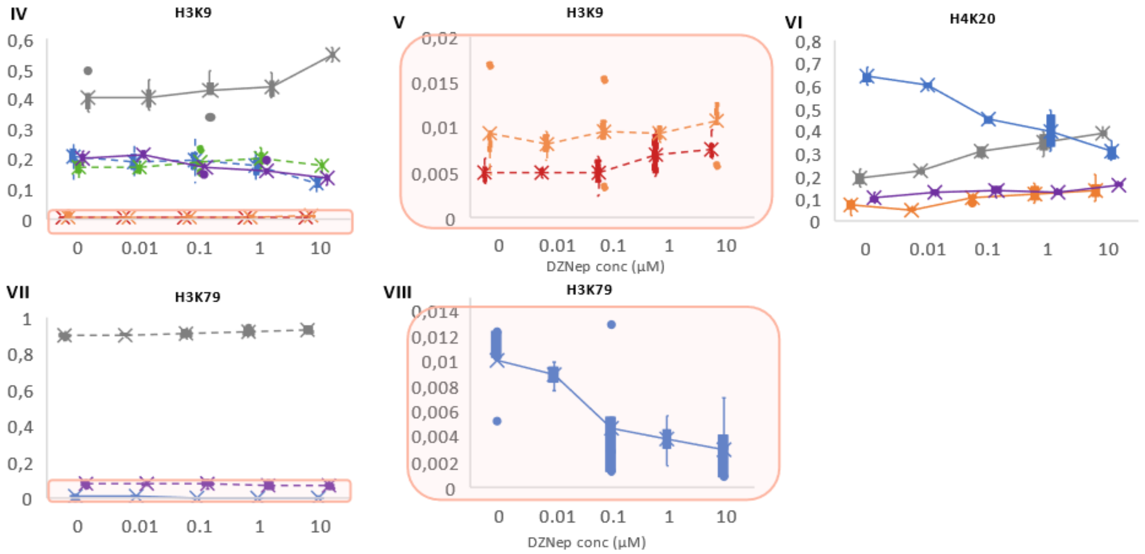

PenG

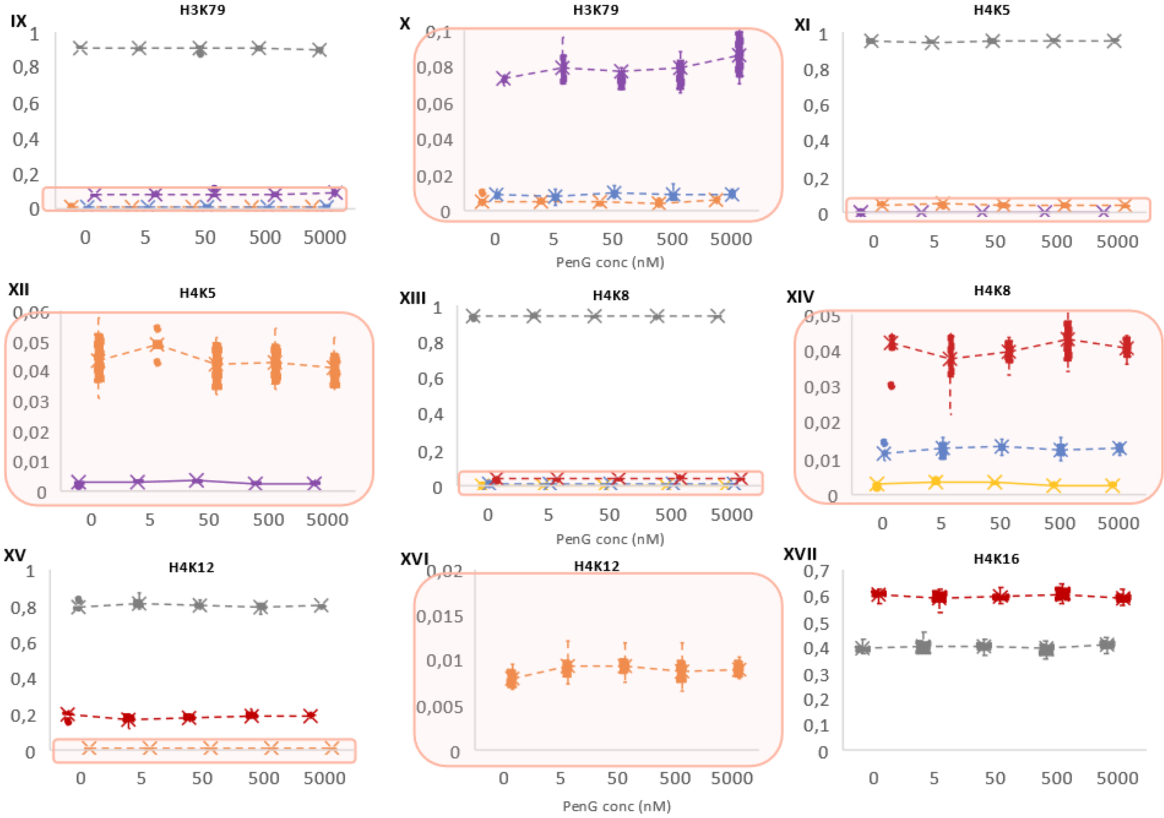

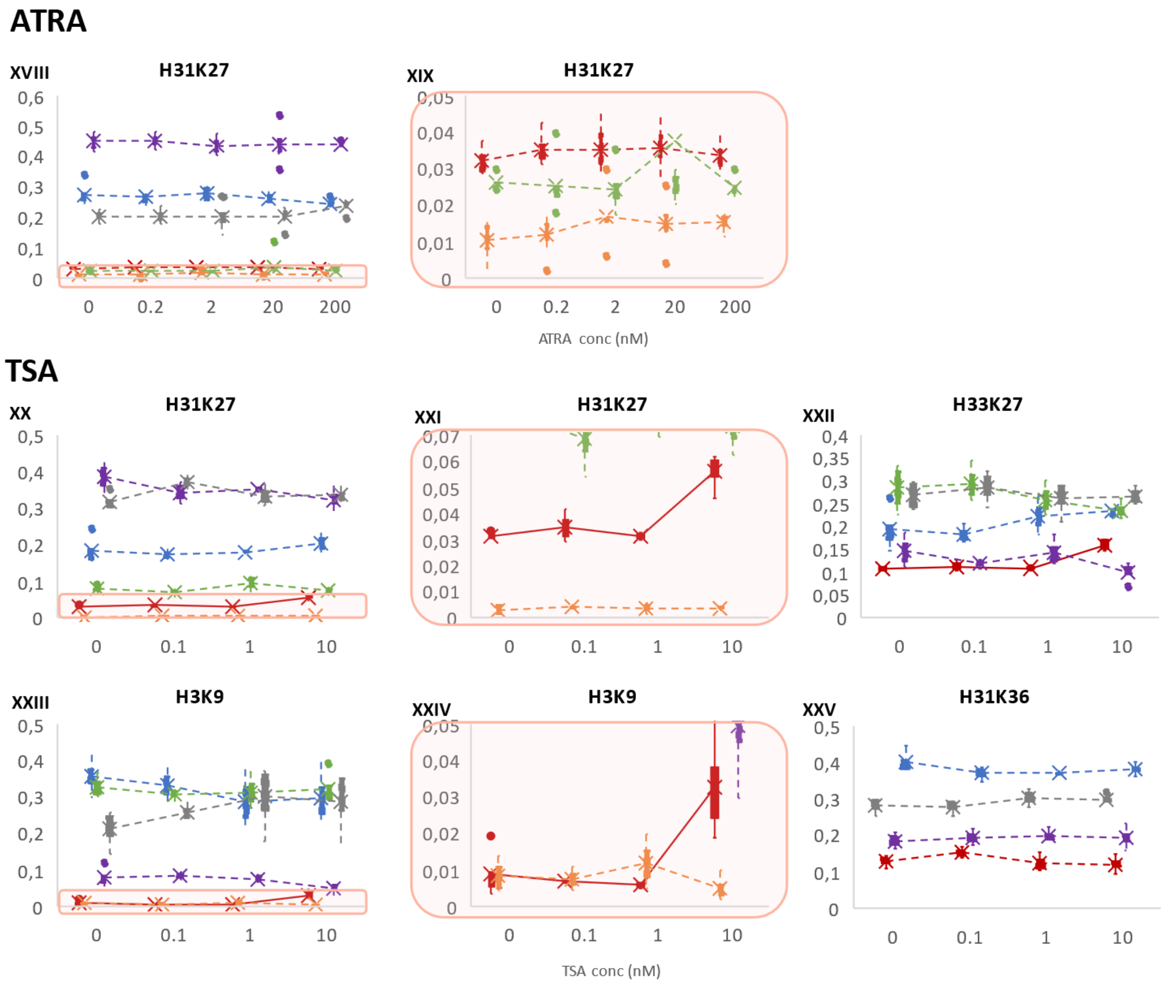

Figure S3. Overview of the relative abundance of all hPTMs discussed in the 'results and discussion' section. Relative abundance in function of increasing concentrations of BIX (nM): (I) H3K9, (II) A zoom with scaled Y-axis of H3K9, (III) H31K27. Relative abundance in function of increasing concentrations of DZNep (µM): (IV) H3K9, (V) A zoom with scaled Y-axis of H3K9, (VI) H4K20, (VII) H3K79, (VIII) A zoom with scaled Y-axis of H3K79. Relative abundance in function of increasing concentrations of PenG (µM): (IX) H3K79, (X) A zoom with scaled Y-axis of H3K79, (XI) H4K5, (XII) A zoom with scaled Y-axis of H4K5, (XIII) H4K8, (XIV) A zoom with scaled Y-axis of H4K8, (XV) H4K12, (XVI) A zoom with scaled Y-axis of H4K12, (XVII) H4K16. Relative abundance in function of increasing concentrations of ATRA (nM): (XVIII) H31K27, (XIX) A zoom with scaled Y-axis of H31K27. Relative abundance in function of increasing concentrations of TSA (nM): (XX) H31K27, (XXI) A zoom with scaled Y-axis of H31K27, (XXII) H33K27, (XXIII) H3K9, (XXIV) A zoom with scaled Y-axis of H3K9, (XXV) H31K36. The represented hPTMs are acetyl (●), formyl (●), crotonyl (●), trimethyl (●), dimethyl (●), monomethyl/butyryl (●), and the unmodified form (●). hPTMs that are significantly changing i.e. with a P-value < 0.05, between one or more concentrations are depicted by full lines, while dotted lines represent statistically stable hPTMs. Note that histone variants H3.1 and H3.3 can only be distinguished by the peptide H3K27-40.
